# Prescription Dispensing for Insulin Glargine After Interchangeable Biosimilar Designation

**DOI:** 10.1001/jamahealthforum.2025.0033

**Published:** 2025-05-02

**Authors:** Stephen J. Murphy, Nicholas C. Holtkamp

**Affiliations:** 1 US Department of Health and Human Services, Washington, DC

## Abstract

**Importance:**

The first US Food and Drug Administration–approved interchangeable biosimilar designation—that for insulin glargine—occurred in 2021, enabling pharmacy substitution for the branded originator. However, the impacts of this interchangeable designation on prescription dispensing are unknown.

**Objective:**

To assess impacts of the transition of Semglee to interchangeable designation on prescription dispensing.

**Design and Setting:**

This economic evaluation analyzed changes in insulin glargine dispensing before and after the introduction of the interchangeable designation using data collected from IQVIA’s National Prescription Audit, a nationally representative comprehensive database of pharmacy dispensing for the US, and PayerTrak. Data cover the time period from September 2019 through June 2024 and were analyzed from June 2023 to December 2024.

**Exposure:**

Any medical diagnosis that would make insulin glargine a relevant treatment.

**Main Outcomes and Measures:**

The primary outcomes were monthly US aggregate pharmacy dispensing of Semglee and insulin glargine-yfgn, measured both in prescription counts (in thousands of prescriptions) and as a proportion of the US aggregate insulin glargine market. Results were disaggregated into Semglee and insulin glargine-yfgn to show that changes in dispensing were associated with the interchangeable designation even after accounting for Semglee’s formulary changes. This evaluation additionally examined dispensing channel and payer type.

**Results:**

After the introduction of interchangeable Semglee and insulin glargine-yfgn in November 2021, there was a discontinuous increase in aggregate Semglee/insulin glargine-yfgn dispensing of 47.41 (95% CI, 19.45-75.38; *P* = .001), suggesting that the interchangeable designation was associated with substantially increased utilization. In addition, Semglee and insulin glargine-yfgn’s share of the total insulin glargine market matched its dispensing trends, demonstrating that the jump in dispensing was not associated with changes in the market as a whole. When disaggregating by channel, there were also statistically significant increases in all 3 channels: retail (20.27; 95% CI, 2.58-37.95; *P* = .03), mail (6.63; 95% CI, 3.58-9.67; *P* < .001), and long-term care (20.52; 95% CI, 11.06-29.98; *P* < .001). This jump, however, coincided with advantageous formulary changes for Semglee but not insulin glargine-yfgn, the increased utilization of which was still associated with the interchangeable designation. In the Medicare Part D, Medicaid, and cash channels, insulin glargine-yfgn adoption grew faster than Semglee, reaching higher levels of dispensing in every single period measured after launch.

**Conclusions and Relevance:**

In this economic evaluation, the first US Food and Drug Administration approval of interchangeable status was associated with increased dispensing of the follow-on. This suggests that interchangeability designation may play an important role in decreasing costs and increasing access to biosimilar prescription drugs for patients.

## Key Points

**Question:**

What are the policy impacts of the US Food and Drug Administration’s interchangeable biosimilar regulatory designation on drug utilization?

**Findings:**

In this economic evaluation, comprehensive US prescription insulin glargine data were evaluated to show that the first interchangeable biosimilar designation—that for insulin glargine in 2021—was associated with an increase in prescription dispensing of the biosimilar.

**Meaning:**

These findings suggest that the US Food and Drug Administration’s interchangeable biosimilar designation can create cost savings to the medical system by increasing substitution to competitor products of the branded originator.

## Teaser

This economic evaluation assesses impacts of the transition of Semglee to interchangeable designation on prescription dispensing.

## Introduction

Recently, there has been a rapidly increasing share of drug spending that is attributable to biologic drugs.^1^ This is predominately due to considerably higher prices for biologics than for small-molecule drugs.^2^ In response, there has been increased emphasis on regulatory mechanisms that govern competitive follow-on entry to branded originator biologics via the biosimilar and interchangeable approval pathways.^3,4,5,6,7,8,9^ Moreover, markets for insulin, one of the most common biologics, generally have faced substantial public health policy attention and scrutiny for their disproportionately high prices in the US and concomitant concerns over accessibility.^10,11^

This article studies the first US Food and Drug Administration (FDA) approval and launch of an interchangeable competitor to a biologic drug—insulin glargine.^12^ This interchangeability designation enables pharmacy substitution of interchangeable biosimilars for branded originators, which may decrease costs and increase access. Additionally, it may increase physician and patient trust in the newly interchangeable biosimilar. It was approved for Semglee—the follow-on competitor to the originator insulin glargine—by the FDA in July 2021. We provide early evidence on the effectiveness of the interchangeable designation in augmenting market competition.

## Methods

### Study Design and Participants

This economic evaluation used prescription drug data from 2 proprietary IQVIA databases: National Prescription Audit (NPA) and PayerTrak. NPA provides representative and near-universal coverage of pharmacy dispensing to patients across the retail (92% coverage), mail (72% coverage), and long-term care (LTC; 78% coverage) pharmacy channels. PayerTrak is similar to NPA but only covers the retail channel (92% coverage); however, it disaggregates by payer type including cash, commercial, Medicaid, and Medicare Part D segments. Each database allows separate measurement of 2 versions of interchangeable insulin, brand-name Semglee and biosimilar insulin glargine-yfgn, vs the remainder of the market.

This study did not require informed consent because it used deidentified and aggregated data in accordance with the Common Rule (45 CFR §46). This study followed the Enhancing the Quality and Transparency of Health Research (EQUATOR) reporting guidelines.

### Study Outcomes

The main outcome was the monthly volume of prescription dispensing of Semglee and insulin glargine-yfgn, expressed in both prescription counts (in thousands of prescriptions) and as a percentage of the total insulin glargine market. This is also disaggregated by dispensing channel or by payer segment. Data cover the period from September 2019 through June 2024. Summary statistics for these data are provided in the Table.

**Table.** Summary Statistics for Pharmacy Dispensing of Semglee and Insulin Glargine-yfgn

Variable	Mean (SD)		
	Semglee		Insulin glargine-yfgn
	Before insulin glargine-yfgn launch	After insulin glargine-yfgn launch	
**Pharmacy dispensing by channel** ^a^			
Prescriptions, thousands	33.61 (20.58)	51.73 (9.10)	111.24 (28.71)
Retail	15.02 (8.27)	40.30 (9.65)	49.36 (18.76)
Long-term care	18.40 (12.47)	3.75 (5.88)	61.36 (13.00)
Mail	0.18 (0.11)	7.68 (2.12)	0.52 (0.15)
Prescription share of insulin glargine market,%	2.1 (1.3)	3.3 (0.6)	7.1 (1.1)
Retail	1.2 (0.7)	3.3 (0.8)	4.0 (1.5)
Long-term care	7.4 (4.9)	1.5 (2.4)	23.4 (5.0)
Mail	0.2 (0.1)	9.2 (2.4)	0.6 (0.2)
Price per 100 IUs, $	11.55 (0.07)	30.78 (4.87)	10.68 (1.73)
Retail	11.53 (0.06)	30.71 (4.91)	10.97 (1.69)
Long-term care	11.60 (0.10)	29.20 (5.68)	10.00 (2.12)
Mail	11.58 (0.09)	31.33 (3.86)	10.98 (1.69)
Price relative to rest of market, %	36.4 (0.2)	114.3 (39.1)	37.7 (2.1)
Retail	36.5 (0.2)	112.5 (38.0)	38.3 (2.3)
Long-term care	36.9 (0.7)	141.9 (71.8)	42.9 (5.1)
Mail	35.6 (0.3)	107.4 (29.5)	36.2 (1.2)
R**etail pharmacy dispensing by payer type**^b^			
Prescriptions, thousands	15.05 (8.28)	40.38 (9.67)	49.45 (18.80)
Cash	0.47 (0.24)	0.21 (0.15)	1.61 (0.64)
Commercial	1.63 (1.07)	32.76 (9.22)	19.21 (8.09)
Medicaid	12.31 (6.67)	3.27 (2.60)	23.98 (8.63)
Medicare Part D	0.63 (0.50)	4.16 (3.22)	46.66 (2.94)
Prescription share of insulin glargine market, %	1.9 (0.7)	3.3 (0.8)	4.1 (1.5)
Cash	6.7 (3.6)	2.9 (2.2)	21.1 (7.5)
Commercial	0.4 (0.2)	7.6 (2.2)	4.4 (1.9)
Medicaid	4.1 (2.2)	1.2 (0.9)	8.6 (3.1)
Medicare Part D	0.1 (0.1)	0.8 (0.6)	0.9 (0.6)
Co-payment per 100 IUs (excluding cash segment), $	0.42 (0.04)	2.20 (0.46)	1.2 (0.2)
Commercial	2.22 (0.52)	2.52 (0.31)	2.52 (0.30)
Medicaid	0.10 (0.04)	0.05 (0.03)	0.04 (0.02)
Medicare Part D	1.20 (0.37)	1.80 (0.52)	1.22 (0.32)

Abbreviation: IUs, international units.

^a^ Pharmacy prescription dispensings of Semglee and insulin glargine-yfgn as measured from IQVIA’s National Prescription Audit database, which covers pharmacy prescribing from retail, mail, and long-term care pharmacies and includes prescription counts as well as the market share of the relevant insulin glargine market inclusive of the branded products. It also includes average pricing data for the retail and long-term care channels but not the mail channel, which must instead be imputed from the retail channel. This data sample covers the period from September 2019 to June 2024.

^b^ Pharmacy prescription dispensing of Semglee and insulin glargine-yfgn as measured from IQVIA’s PayerTrak, which covers only the retail pharmacy channel but includes disaggregation by patient insurance payer type (cash, commercial, Medicaid, and Medicare Part D). While PayerTrak does not include pricing data, it does include average co-payment data except for the cash segment, where it is undefined. The PayerTrak data sample covers the period from July 2020 to June 2024.

### Statistical Analysis

This analysis primarily evaluated trends in interchangeable insulin utilization using observational data. We examined changes in dispensing after the interchangeable versions of Semglee and insulin glargine-yfgn were introduced in November 2021 using interrupted time-series analysis where we compared the slopes and intercepts of linear models for the combination of Semglee and insulin glargine-yfgn via Stata’s ITSA package (StataCorp), utilizing generalized linear models and Newey-West standard errors adjusted for 2 lags to address potential autocorrelation. Additionally, we used panel regression comparing the slope and level parameters for the first 14 months (the number of months Semglee was a biosimilar before the relaunch as interchangeable) after launch of Semglee and insulin glargine-yfgn (eMethods in Supplement 1). Secondary analyses disaggregated by channel and payer type.

Data analysis occurred from June 2023 to December 2024 using Stata MP, version 17.0 (StataCorp), statistical software. *P* values were 2-sided with a significance threshold of <.01.

## Results

**Figure 1.**
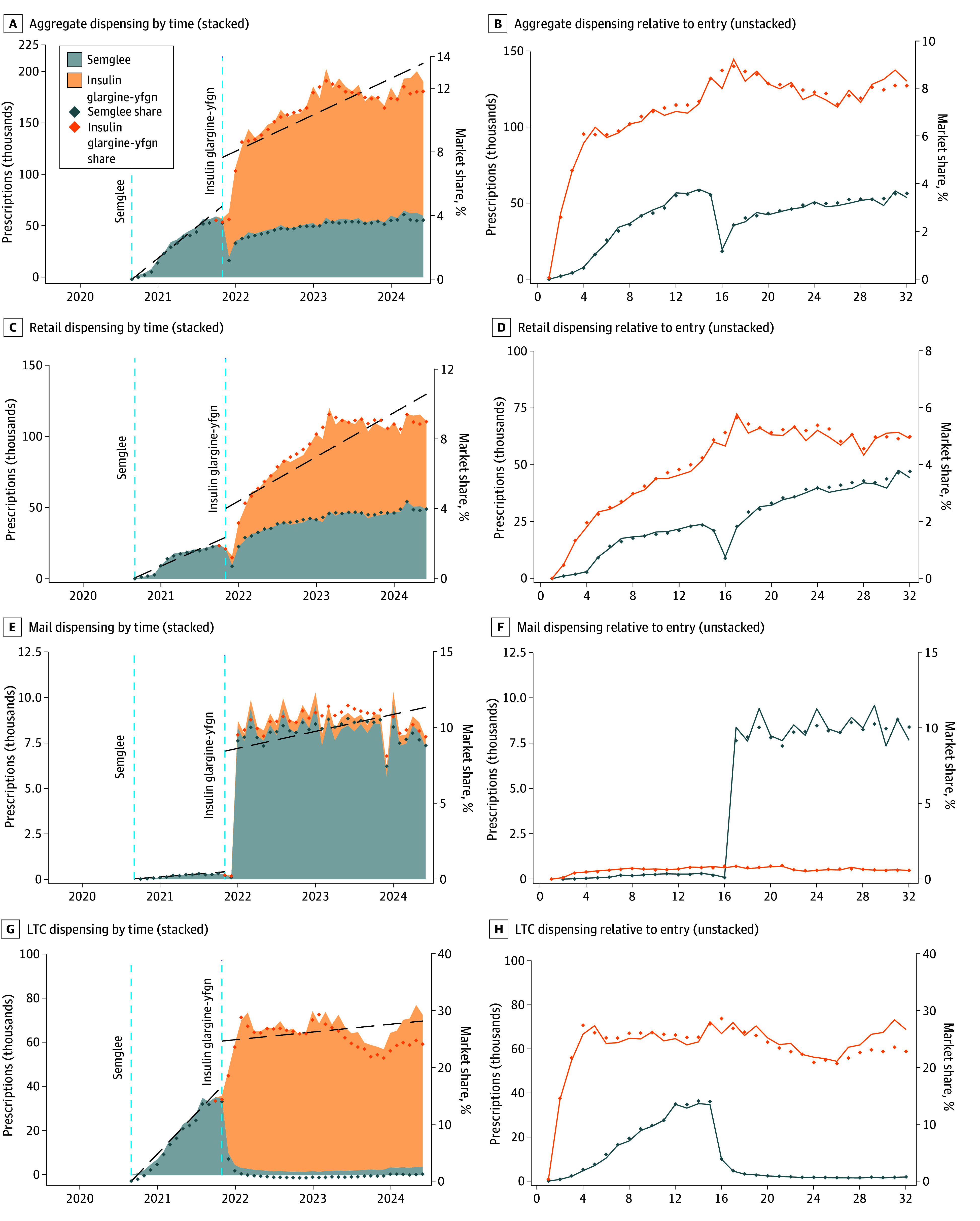
Semglee and Insulin Glargine-yfgn Pharmacy Dispensing Volumes by Channel The left column (A, C, E, and G) displays prescribing of Semglee and insulin glargine-yfgn plotted by calendar time stacked on top of one another to allow for visualizing the combined trends as well as linear interrupted time-series models (dashed lines). The right column (B, D, F, and H) plots the data based on months relative to launch date for each product and is unstacked to facilitate direct comparison of launch dynamics between Semglee and insulin glargine-yfgn. Because the left column stacks insulin glargine-yfgn on top of Semglee, whereas the right column unstacks them, insulin glargine-yfgn share is actually the sum of Semglee and insulin glargine-yfgn. Additionally, Semglee was only a biosimilar at its original launch, whereas during the entry of interchangeable insulin glargine-yfgn, Semglee was relaunched as an interchangeable as well. Data are from IQVIA’s National Prescription Audit database covering September 2019 through June 2024. LTC indicates long-term care.

Changes in biosimilar insulin dispensing were evaluated after the introduction of the interchangeable products using interrupted time-series models. Figure 1A shows that after the introduction of interchangeable Semglee and insulin glargine-yfgn in November 2021, there was a discontinuous increase in aggregate Semglee/insulin glargine-yfgn dispensing of 47.41 (95% CI, 19.45-75.38; *P* = .001), suggesting that the interchangeable designation was associated with substantially increased utilization. In addition, Semglee and insulin glargine-yfgn’s share of the total insulin glargine market matched its dispensing trends, demonstrating that the jump in dispensing was not associated with changes in the market as a whole. When disaggregating by channel, there were also statistically significant increases in all 3 channels: retail (20.27; 95% CI, 2.58-37.95; *P* = .03), mail (6.63; 95% CI, 3.58-9.67; *P* < .001), and long-term care (20.52; 95% CI, 11.06-29.98; *P* < .001). This jump, however, coincided with advantageous formulary changes for Semglee (due to a new higher price/rebate structure^13^), which also may have influenced dispensing. Figure 1 therefore additionally separates dispensing between Semglee and insulin glargine-yfgn, the latter of which was not similarly influenced by formulary changes. Insulin glargine-yfgn itself also experienced a discontinuous jump in dispensing during this transition time despite competing against newly interchangeable Semglee that was advantaged by pharmacy benefit manager formularies—again suggesting that the interchangeability designation was associated with increases in dispensing.

Further evidence that the interchangeability designation was associated with increased utilization is shown by formally comparing dispensing of Semglee and insulin glargine-yfgn separately measured in months since original entry (Figure 1B) using linear panel regressions applied to the number of months (14) that Semglee was active preinterchangeability (and formulary advantages). In the first 14 months after launch, both the slope and intercept parameters for insulin glargine-yfgn were larger than those for Semglee (6.15 vs 5.12 and 43.91 vs −7.19), but only the intercept was statistically significantly larger (*P* = .02). This highlights the immediate acceleration in insulin glargine-yfgn dispensing on launch, which was substantially faster than the more gradual initial adoption of Semglee. Moreover, insulin glargine-yfgn reached peak levels of dispensing over twice as high as Semglee (approximately 8.9% vs 4.1% of all insulin glargine prescriptions).

When disaggregating results by distribution channel, for retail and LTC channels, where automatic substitution at the pharmacy may play a meaningful role, insulin glargine-yfgn dispensing experienced an immediate jump. Panel regression models for the initial 14 months after launch demonstrated a statistically significant larger slope parameter for insulin glargine-yfgn than Semglee in the retail channel (3.59 vs 2.03 *P* = .005), and the LTC channel’s insulin glargine-yfgn intercept coefficient was statistically significantly larger at 38.67 vs −5.86 (*P* = .008). In fact, in the LTC channel, insulin glargine-yfgn entirely crowded out Semglee. In the mail channel, on the other hand, insulin glargine-yfgn’s launch dynamics initially achieved approximately 3 times the volume of original Semglee (0.52 vs 0.18; Table), with a statistically significant larger intercept parameter at 0.16 vs −0.04 (*P* = .06). But after formulary changes, the now interchangeable Semglee spiked by a factor of 40 (0.18 vs 7.68; Table) in January 2022, overwhelming insulin glargine-yfgn and the original Semglee trend.

**Figure 2.**
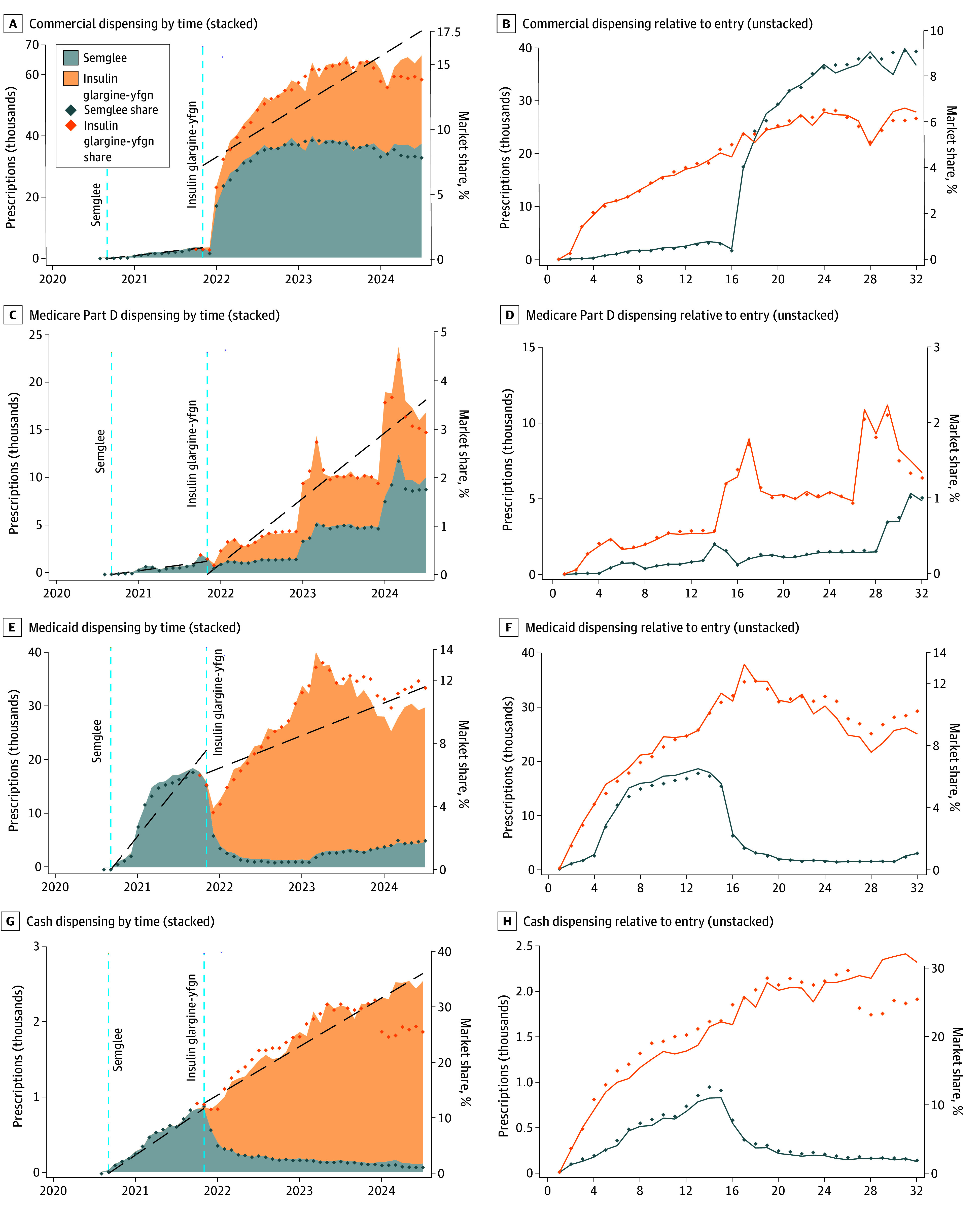
Semglee and Insulin Glargine-yfgn Retail Pharmacy Dispensing Volumes by Payer Type The left column (A, C, E, and G) displays prescribing of Semglee and insulin glargine-yfgn plotted by calendar time stacked on top of one another to allow for visualizing the combined trends as well as linear interrupted time-series models (dashed lines). The right column (B, D, F, and H) plots the data based on months relative to launch date for each product and is unstacked to facilitate direct comparison of launch dynamics between Semglee and insulin glargine-yfgn. Because the left column stacks insulin glargine-yfgn on top of Semglee, whereas the right column unstacks them, insulin glargine-yfgn share is actually the sum of Semglee and insulin glargine-yfgn in the left column. Additionally, Semglee was only a biosimilar at its original launch, whereas during the entry of interchangeable insulin glargine-yfgn, Semglee was relaunched as an interchangeable as well. Data are from IQVIA’s National Prescription Audit database covering September 2019 through June 2024.

Lastly, in Figure 2, results are disaggreated by payer segment for the retail channel. In the Medicare Part D, Medicaid, and cash channels, insulin glargine-yfgn adoption grew faster than Semglee, reaching higher levels of dispensing in every single period measured after launch. Formally, in the initial 14 months after launch, insulin glargine-yfgn had larger slope and intercept parameters than Semglee in each of these 3 segments and had a statistically significantly larger Medicare slope (0.18 vs 0.10; *P* = .10), Medicare intercept (0.57 vs −0.18; *P* = .10), and cash slope parameters (0.11 vs 0.06; *P* = .007). This is especially prevalent in the cash channel, where pharmacy benefit manager formularies would be irrelevant and, hence, cannot conflate the multiple potential drivers of increased uptake. The commercial segment’s results are more complex. Insulin glargine-yfgn still initially grew substantially faster than Semglee on launch until January 2022, when Semglee dispensing spiked, surpassing that of insulin glargine-yfgn. Formally, the initial 14-month postlaunch models show a statistically significant larger slope parameter (1.34 vs 0.26; *P* ≤ .001). However, beyond this period of initial launch dynamics, Semglee dispensing spiked above insulin glargine-yfgn coincidentally with formulary changes—hence, it is not possible to perfectly disentangle this from the possible effect of interchangeability designation, but it highlights evidence that suggests both mechanisms may have been associated with enhanced dispensing.

## Discussion

We provide novel evidence that the transition of Semglee from follow-on to interchangeable status is strongly associated with the follow-on’s market expansion—substantially larger than the original introduction of Semglee—but with heterogeneity along channel and payer segment that help elucidate the underlying mechanisms of action. Other work has highlighted the initial rise in interchangeable insulin glargine dispensing after the introduction of the interchangeable designation.^14^ This work builds on theirs by showing that this initial increase in dispensing was not associated solely with concurrent formulary changes and has persisted years after the interchangeability designation.

The present results illustrate that interchangeability may be valuable for inducing increased utilization of biosimilars, which in turn may lead to cost savings and increased patient access. In the case of insulin glargine, which is primarily distributed through retail pharmacies, this may work through automatic pharmacy substitution or increased physician prescribing if the interchangeability designation generates more trust in the biosimilar. Other biosimilars may similarly experience increased utilization if deemed interchangeable, although their experience may vary along a number of dimensions, for instance due to nature of administration, existing competitors, and formulary strategies.

### Limitations

While we observed manufacturer prices, we were unable to directly observe rebates, which limits our ability to estimate cost savings due to the increase utilization of biosimilar insulin glargine. Moreover, we were unable to directly observe formulary design for patients filling prescriptions.

## Conclusions

This economic evaluation shows that the biosimilar and interchangeable biosimilar markets are rapidly evolving alternatives to originator biologics. We provide novel evidence that the FDA approval of Semglee as an interchangeable biosimilar was associated with increased dispensing of biosimilar insulin glargine. This shows that interchangeability may be valuable for lowering costs and increasing patient access for costly biologics.

## Notes

Supplement 1.
eMethods

Supplement 2.
Data Sharing Statement
