# Supplementary material for: Prescription Dispensing for Insulin Glargine After Interchangeable Biosimilar Designation
Source: JAMA Health Forum. 2025 May 2;6(5):e250033. doi: 10.1001/jamahealthforum.2025.0033 (PMC12048848; doi:10.1001/jamahealthforum.2025.0033)
Supplement: Supplement 1. — eMethods [file jamahealthforum-e250033-s001.pdf]

Supplemental Online Content

Murphy SJ, Holtkamp NC. Prescription dispensing for insulin glargine after interchangeable biosimilar designation. *JAMA Health Forum*. 2025;6(5):e250033. doi:10.1001/jamahealthforum.2025.0033

eMethods

This supplemental material has been provided by the authors to give readers additional information about their work.

© 2025 Murphy SJ et al. *JAMA Health Forum*.

eMethods

Statistical Analysis:

Our analysis primarily evaluates trends in interchangeable insulin utilization using observational data. We focus on the introduction of the interchangeable designation by examining changes in dispensing after the interchangeable versions of Semglee and insulin glargine-yfgn are introduced in November 2021. Our analytics are presented in the body of the manuscript via two multi-panel figures. Figure 1 uses IQVIA NPA data which provides pharmacy dispensing data from Retail, Long-Term Care and Mail channels, and Figure 2 uses IQVIA PayerTrak data which provides pharmacy dispensing data only from the Retail channel, but breaks it down by payer types for the Commercially insured, Medicare D enrollees, Medicaid enrollees, and Cash paying patients separately. Each figure is presented in two columns with analogous structures. The left-hand column provides time-series data showing the trends in prescription dispensing of Semglee and insulin glargine-yfgn measured through calendar time, with the insulin glargine-yfgn data stacked on top of Semglee (which enters first). Marked by vertical dashed lines are both the entry point of the original biosimilar (non-interchangeable) Semglee, and the transition point where interchangeable insulin glargine-yfgn entered and Semglee introduced a new interchangeable version simultaneously. By stacking the data via a time-series area plot, we show calendar time trends in either Semglee or insulin glargine-yfgn separately, as well as the combination of the two as they are owned by the same corporation. Moreover, we estimate interrupted time series models for the combination of Semglee and insulin glargine-yfgn using Stata's ITSA package which employs generalized linear models and Newey-West standard errors with lag terms to handle the potential for autocorrelation. These models' coefficients are presented below in Table A1, and they are also projected on top of the raw data in the figure in the body of the manuscript.

The right-hand column of each of these figures then provides a related analysis but plots the time-series trends of each of Semglee and insulin glargine-yfgn measured as months relative to each product's original launch rather than calendar time. This highlights the relative rates of adoption of Semglee and insulin glargine-yfgn post launch, providing insight into the potential impact of the interchangeability designation. Note in these figures in the right-hand column the data for Semglee and insulin glargine-yfgn are unstacked and presented as line graphs rather than as area plots, hence their levels are directly

© 2025 Murphy SJ et al. *JAMA Health Forum*.

comparable. This additionally lends itself to estimating linear panel data models that are roughly analogous to our earlier ITS models—but instead of comparing parameters for one series (the combination of Semglee and insulin glargine-yfgn) between two time periods, we instead compare two separate groups (Semglee vs. insulin glargine-yfgn for the same time period since initial launch. To do this, we use the Newey command to estimate linear panel models that are robust to autocorrelation, with separate slope and level parameters for each of Semglee and insulin glargine-yfgn for the first 14 months of data post launch for each drug. We use the first 14 months post launch because that is how many months of Semglee activity there is before insulin glargine-yfgn launches and Semglee relaunches as an interchangeable and then faces advantageous formulary placement. Hence, that is a highly relevant direct comparison that is not affected by other major confounding forces. The level and slope parameters are reported in the bottom panel of Table A1 below, as well as with 95% confidence intervals and F-statistics generated from Wald tests for the difference of each pair of coefficients along with the p-value of the test.

© 2025 Murphy SJ et al. *JAMA Health Forum*.

Table A1: Regression Coefficients, Confidence Intervals, and Statistical Tests.

ITS Models	All Pharmacies	Retail Pharmacies					LTC Pharmacies	Mail Pharmacies
		All Payers	Commercial	Medicare D	Medicaid	Cash		
Baseline Level	-2.07	0.74	-0.35**	-0.13	-0.47	-0.02**	-2.80**	0.02
(95% CI)	(-6.09, 1.95)	(-2.56, 4.03)	(-0.63, -0.07)	(-0.33, 0.06)	(-3.24, 2.29)	(-0.05, -0.00)	(-5.10, -0.49)	(-0.01, 0.05)
Baseline Slope	5.12***	2.03***	0.25***	0.10***	1.59***	0.06***	3.06***	0.03***
(95% CI)	(4.63, 5.62)	(1.64, 2.43)	(0.22, 0.28)	(0.06, 0.14)	(1.25, 1.94)	(0.06, 0.06)	(2.79, 3.33)	(0.02, 0.03)
Change in Level	47.41***	20.27**	26.90***	-1.37*	-4.16	0.07	20.52***	6.63***
(95% CI)	(19.45, 75.38)	(2.58, 37.95)	(12.42, 41.38)	(-2.96, 0.22)	(-10.45, 2.13)	(-0.06, 0.20)	(11.06, 29.98)	(3.58, 9.67)
Change in Slope	-2.16**	0.56	1.13***	0.47***	-1.09***	-0.01**	-2.77***	0.05
(95% CI)	(-3.82, -0.50)	(-0.53, 1.65)	(0.44, 1.81)	(0.34, 0.61)	(-1.57, -0.60)	(-0.02, -0.00)	(-3.39, -2.15)	(-0.10, 0.20)

Launch Comparison	All Pharmacies	Retail Pharmacies					LTC Pharmacies	Mail Pharmacies
		All Payers	Commercial	Medicare D	Medicaid	Cash		
Semglee Level	-7.19***	-1.30	-0.46***	-0.18	-0.62	-0.03**	-5.86***	-0.04**
(95% CI)	(-12.01, -2.37)	(-5.23, 2.63)	(-0.70, -0.22)	(-0.43, 0.07)	(-4.53, 3.28)	(-0.06, -0.01)	(8.63, -3.09)	(-0.07, -0.00)
YFGN Level	43.91**	5.08	1.51	0.57	2.84	0.20	38.67**	0.16
(95% CI)	(3.10, 84.73)	(-4.03, 14.20)	(-1.94, 4.97)	(-0.29, 1.43)	(-1.78, 7.45)	(-0.09, 0.49)	(6.81, 70.52)	(-0.04, 0.37)
F-Statistic (P-Value)	6.59 (0.02)**	1.76 (0.20)	1.39 (0.25)	2.94 (0.10)*	1.39 (0.25)	2.78 (0.11)	8.26 (0.01)***	3.88 (0.06)*
Semglee Slope	5.12***	2.03***	0.26***	0.10***	1.61***	0.06***	3.06***	0.03***
(95% CI)	(4.58, 5.66)	(1.60, 2.46)	(0.24, 0.29)	(0.06, 0.15)	(1.15, 2.06)	(0.06, 0.07)	(2.77, 3.36)	(0.02, 0.03)
YFGN Slope	6.15***	3.59***	1.34***	0.18***	1.97***	0.11***	2.51	0.04***
(95% CI)	(2.02, 10.27)	(-5.23, 2.63)	(0.99, 1.69)	(0.06, 0.15)	(1.47, 2.47)	(0.08, 0.14)	(-0.68, 5.70)	(0.02, 0.06)
F-Statistic (P-Value)	0.26 (0.62)	9.46 (0.01)***	39.30 (0.00)***	3.02 (0.10)*	1.21 (0.28)	8.74 (0.01)***	0.13 (0.73)	1.45 (0.24)

*p<=0.10, **p<=0.05, ***p<=0.01

Table A1 displays the point estimates and 95% confidence intervals of our underlying regression models. It is broken into two panels: the top panel reports the results of our Interrupted Times Series analysis of aggregate utilization (the sum of Semglee and insulin glargine-yfgn through time before and after the introduction of insulin glargine-yfgn and the interchangeable relaunch of Semglee. It utilizes a linear form estimated with the Stata ITSA package using GLM and Newey-West standard errors adjusted for 2 lags. We report the baseline level and slope parameters for the pre-period, as well as the change in level and change in slope parameters. The bottom panel uses Stata's Newey regression estimator to estimate level and slope parameters in a panel model of prescription utilization for each of Semglee and insulin glargine-yfgn for the first 14 months post launch to compare their launch dynamics. It also presents the associated F-statistics generated from Wald tests of the difference in level and the difference in slope parameter.

© 2025 Murphy SJ et al. *JAMA Health Forum*.
