# Supplementary material for: Prescription Dispensing for Insulin Glargine After Interchangeable Biosimilar Designation
Source: JAMA Health Forum. 2025 May 2;6(5):e250033. doi: 10.1001/jamahealthforum.2025.0033 (PMC12048848; doi:10.1001/jamahealthforum.2025.0033)
Supplement: Supplement 2. — Data Sharing Statement [file jamahealthforum-e250033-s002.pdf]

Data Sharing Statement

Murphy. Prescription Dispensing for Insulin Glargine After Interchangeable Biosimilar Designation. *JAMA Health Forum*. Published May 02, 2025. doi:10.1001/jamahealthforum.2025.0033

Data
Data available: No

Additional Information
Explanation for why data not available: We use proprietary IQVIA data that contractually cannot be shared.
